# Supplementary material for: Cells released from S. epidermidis biofilms present increased antibiotic tolerance to multiple antibiotics
Source: PeerJ. 2019 May 15;7:e6884. doi: 10.7717/peerj.6884 (PMC6525591; doi:10.7717/peerj.6884)
Supplement: Table S1 [file peerj-07-6884-s001.docx]

**Supplementary Table S1.** Mechanisms of action and peak serum concentrations (PSC) in mg/L of the 9 antibiotics used in this study

| Mechanism of action ^a^ | Antibiotic | PSC (mg/L) |
| --- | --- | --- |
| Cell wall synthesis inhibitor | Dicloxacillin | **59**  (Friberg, O.; Jones, I.; Sjöberg, L.; Söderquist, B.; Vikerfors, T. & Källman, J., 2004. Antibiotic concentrations in serum and wound fluid after local gentamicin or intravenous dicloxacillin prophylaxis in cardiac surgery. *Scand J Infect Dis.*, 35(4):251-254) |
|  | Teicoplanin | **50**  (Mensa, J., 1998. *Guía terapéutica antimicrobiana* 8th ed., Masson) |
|  | Vancomycin | **40**  (National Committee for Clinical Laboratory Standards, 1997. *Methods for dilution: antimicrobial susceptibility tests for bacteria that grow aerobically - Fifth Edition: Approved Standard M7-A5*, Wayne, PA, USA: NCCLS) |
| Nucleic acids synthesis inhibitor | Ciprofloxacin | **4.5**  (Bayer HealthCare Pharmaceuticals Inc., 2004. CIPRO (R) (ciprofloxacin hydrochloride) Tablets, pp.1–31 ) |
|  | Rifampicin | **10**  (National Committee for Clinical Laboratory Standards, 1997. *Methods for dilution: antimicrobial susceptibility tests for bacteria that grow aerobically - Fifth Edition: Approved Standard M7-A5*, Wayne, PA, USA: NCCLS) |
| Protein synthesis inhibitor | Erythromycin | **10**  (Bennett, J.E.; Dolin, R. & Blaser, M.J., 2014. Basic principles in the diagnosis and management of infectious diseases. In *Principles and Practice of Infectious Diseases,* pp. 358–376, Philadelphia, PA, Elsevier Saunders) |
|  | Gentamicin | **10**  (Demczar, D.J.; Nafziger, A.N. & Bertino, J.S., 1997. Pharmacokinetics of gentamicin at traditional versus high doses: Implications for once-daily aminoglycoside dosing. *Antimicrobial Agents and Chemotherapy*, 41(5), pp.1115–1119) |
|  | Linezolid | **18**  (Prydal, J.I.; Jenkins, D.R.; Lovering, A. & Watts, A., 2005. The pharmacokinetics of linezolid in the non-inflamed human eye. *The British journal of ophthalmology*, 89(11), pp.1418–9) |
|  | Tetracycline | **16**  (National Committee for Clinical Laboratory Standards, 1997. *Methods for dilution: antimicrobial susceptibility tests for bacteria that grow aerobically - Fifth Edition: Approved Standard M7-A5*, Wayne, PA, USA: NCCLS) |

^a^ The mechanism of action of the antibiotics was determined by the information sheet provided by the antibiotics manufacturer.
