# Supplementary material for: Cells released from S. epidermidis biofilms present increased antibiotic tolerance to multiple antibiotics
Source: PeerJ. 2019 May 15;7:e6884. doi: 10.7717/peerj.6884 (PMC6525591; doi:10.7717/peerj.6884)
Supplement: Table S2 [file peerj-07-6884-s002.docx]

**Supplementary Table S2.** Determination of the MIC ranges, in mg/L, of 9 antibiotics against a planktonic population of *S. epidermidis* 9142 and evaluation, by EUCAST, CLSI, and BSAC standards, of the susceptibility to the antibiotics tested

| Antibiotic | MIC range  (mg/L) | Clinical breakpoint (mg/L) | | Evaluation |
| --- | --- | --- | --- | --- |
|  |  | **S ≤** | **R >** |  |
| Dicloxacillin | 0.125-0.25 | 0.25 | 0.5 | Susceptible |
| Teicoplanin | 2-4 | 4 | 4 | Susceptible |
| Vancomycin | 1-2 | 4 | 4 | Susceptible |
| Ciprofloxacin | 8-16 | 1 | 1 | **Resistant** |
| Rifampicin | 0.004-0.008 | 0.064 | 0.5 | Susceptible |
| Erythromycin | 1 | 1 | 2 | Susceptible |
| Gentamicin | 1-2 | 1 | 1 | **Intermediate** |
| Linezolid | 8 | 4 | 4 | **Resistant** |
| Tetracycline | 0.5 | 1 | 2 | Susceptible |
