# Supplementary material for: Cells released from S. epidermidis biofilms present increased antibiotic tolerance to multiple antibiotics
Source: PeerJ. 2019 May 15;7:e6884. doi: 10.7717/peerj.6884 (PMC6525591; doi:10.7717/peerj.6884)
Supplement: Table S3 [file peerj-07-6884-s003.docx]

**Supplementary Table S3.** Determination of the MIC ranges, in mg/L, of vancomycin in planktonic populations of the 11 distinct *S. epidermidis* isolates (EUCAST breakpoint for Vancomycin: Susceptible if MIC ≤ 4mg/L; Resistant if MIC > 4 mg/L)

| *S. epidermidis* isolate | Vancomycin  MIC range (mg/L) |
| --- | --- |
| RP62A | 2 - 4 |
| 9142 | 1 - 2 |
| IE186 | 1 - 2 |
| PT12003 | 1 - 2 |
| 1457 | 4 |
| DEN69 | 1-2 |
| URU23 | 2 |
| IE214 | 4 |
| PT13032 | 2 |
| ICE09 | 2 |
| MEX60 | 2 |
