# Supplementary figures and images for: Cells released from S. epidermidis biofilms present increased antibiotic tolerance to multiple antibiotics
Source: PeerJ. 2019 May 15;7:e6884. doi: 10.7717/peerj.6884 (PMC6525591; doi:10.7717/peerj.6884)

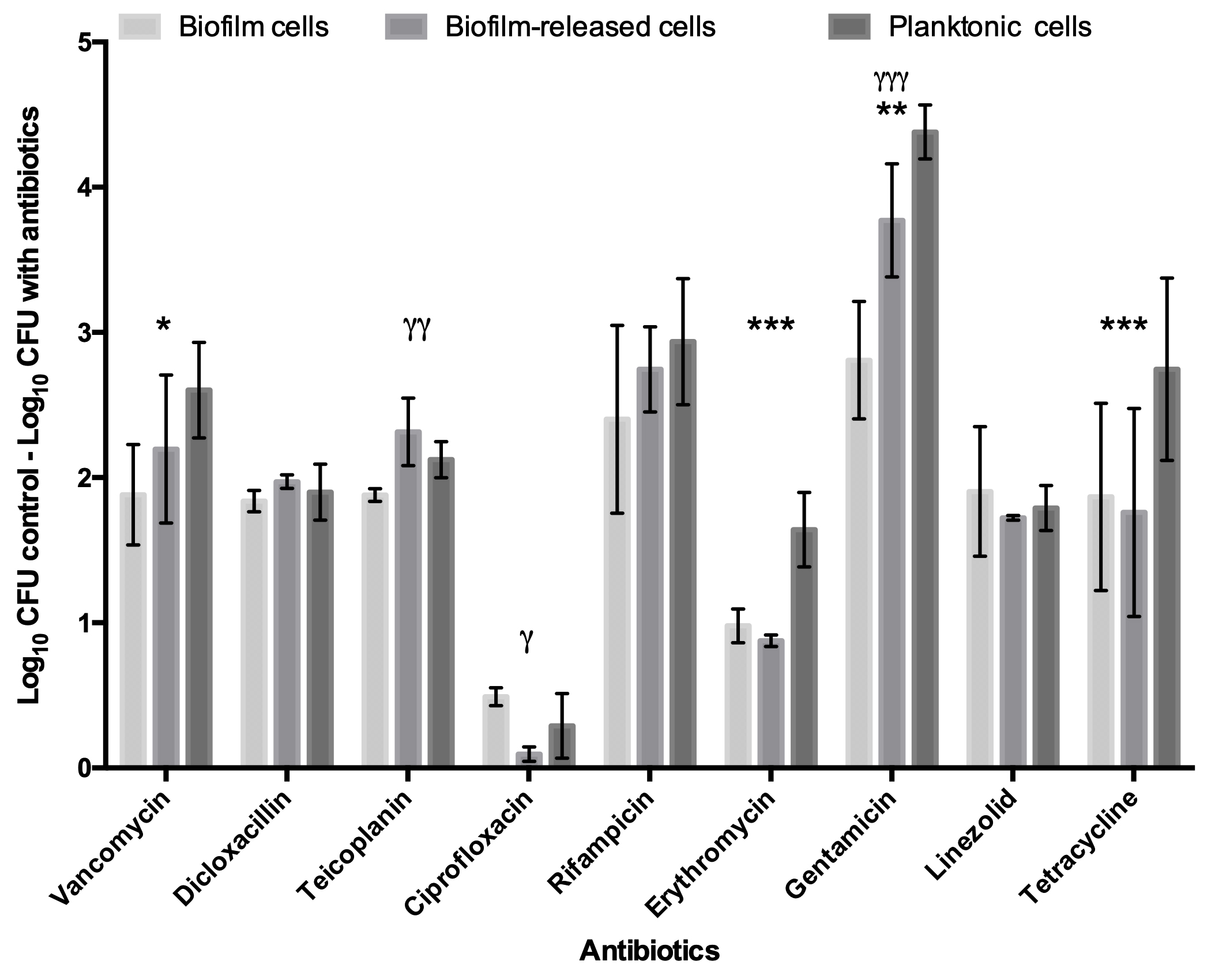

Supplement: Figure S1 — The columns represent the mean plus or minus standard error deviation, of at least three independent experiments. Statistically significant differences between biofilm cells and Brc are represented with * (* p < 0.05; *** p < 0.001) and between Brc and their planktonic counterparts with γ (γ p < 0.05; γ γ p < 0.01; γγ p < 0.001). [file peerj-07-6884-s004.jpg]
